# Supplementary material for: The study on the adsorption characteristics of anthracite under different temperature and pressure conditions
Source: PLoS One. 2025 Mar 11;20(3):e0310863. doi: 10.1371/journal.pone.0310863 (PMC11896062; doi:10.1371/journal.pone.0310863)
Supplement: S1 Table — (DOCX) [file pone.0310863.s001.docx]

Table1 Physicochemical properties of CO_2_, CH_4_ and N_2_

| Gas mode | CO_2_ | CH_4_ | N_2_ |
| --- | --- | --- | --- |
| Dynamic diameter(nm) | 3.30 | 3.80 | 3.64 |
| Critical temperature(K) | 304.29 | 190.45 | 126.15 |
| Critical pressure(MPa) | 7.38 | 4.57 | 3.40 |
| Boiling point(K) | 194.65 | 111.55 | 77.35 |
| Polarizability (10^‒25^ cm^3^) | 26.50 | 26.00 | 17.60 |
