# Supplementary material for: The study on the adsorption characteristics of anthracite under different temperature and pressure conditions
Source: PLoS One. 2025 Mar 11;20(3):e0310863. doi: 10.1371/journal.pone.0310863 (PMC11896062; doi:10.1371/journal.pone.0310863)
Supplement: Attachment — Submitted filename: Comments1.docx [file pone.0310863.s005.docx]

Comments:

This work presented a study on the potential of anthracite coal as an effective medium for adsorbing CO_2_. Although the resulting adsorbent demonstrated excellent adsorption efficiency, there are some corrections that the authors must address:

1. Both the abstract and conclusion parts need to show the innovation of this paper to differentiate the present work from others.

2. What was the main reason for selecting the anthracite coal for this research? It should be mentioned carefully in detail at the beginning of the Introduction section.

3. In section 3.1., it was mentioned that “The larger the polarizability of the gas molecule is, the greater the dispersion force and induction force are. This makes it easier for the gas to occupy the adsorption site on the surface of the”. It means that the higher polarizability of CO_2_ molecules facilitates their adsorption on the adsorbent’s surface compared to other gases. Therefore, it is recommended that the Authors work on the schematic diagram of interactions between anthracite and the desired molecule (i.e., CO_2_) for more clarification.

4. What is the meaning of the “effective stress”? It has been mentioned several times in section 3.1 without any clarification. Please define it before and explain clearly how this factor(s) impacts the gas adsorption capacity of the anthracite used.

5. The language of this paper needs to be improved at the academic level as a whole. Active or passive sentences were not carefully described. The Authors must correct the language errors. Some are:

i. Abstract “The results show that the adsorption capacities of anthracite for these three gases are”. The results showed that...
ii. Section 1 Introduction “He gas had a weak adsorption capacity with an almost negligible volume change”. He is a specific gas. Use an article before that “The He”. It has been repeated many times in the drafts, please correct all.
iii. Section 1 Introduction “However, there are fewer experiments that use columnar coal for analysis, and there is relatively little research on the adsorption micro-mechanism using molecular simulation combined with experiments”. There are fewer experiments that should be changed to “fewer experiments use”.
iv. Section 2.1 “F8 were opened to vacuum the system to discharge any impurities such as air and water from the enture device”. What is the meaning of enture? It might be entire.
v. Section 2.1 “After the free volume test was completed, the system was vacuumized again to ensure that He gas inside the system and the coal sample was completely pumped out to avoid experimental errors”. There is no vocabulary in English for vacuumized. Vacuumed is the correct form.
vi. Section 3.2 “The smaller the radius of the probe molecule is, the larger the pore volume that can be detected is”. The second is should be deleted.
vii. Section 3.3 “To further explore the pore distribution characteristics of the anthracite macromolecular structure, on the basis of obtaining the pore distribution characteristics”. On the basis of should be changed to based on.
