# Supplementary material for: The study on the adsorption characteristics of anthracite under different temperature and pressure conditions
Source: PLoS One. 2025 Mar 11;20(3):e0310863. doi: 10.1371/journal.pone.0310863 (PMC11896062; doi:10.1371/journal.pone.0310863)
Supplement: Attachment — Submitted filename: Review-Saood - PLOS.docx [file pone.0310863.s003.docx]

**Reviewers’ comments**

**Manuscript:** “The study on the adsorption characteristics of anthracite under different temperature and pressure conditions”, has been reviewed.

**Comments:**
Thank you for inviting me to review this manuscript. The paper itself is moderately written, although somewhat less descriptive. The authors have conducted a moderate thorough literature review, undertaken a piece of data collection and have analyze information moderately. The manuscript can be accepted as is, with minor revision. Besides, following correction should be incorporated: -

**General Comments**

**Overall, the paper needs thorough English correction. There are a lot of grammatical mistakes. The authors are requested to get proper English correction.**

Abstract should be improved by adding results, conclusions and other relevant info. Also English needs to be improved in the abstract section.

**Introduction**

In the introduction section what does the Author mean by “stress condition” Please explain.

I suggest to further elaborate more about point of zero charge analysis to know the surface charge and its effect should be included in the methodology and results section. The author can refer these articles https://www.tandfonline.com/doi/full/10.1080/07391102.2023.2186704.

What is the novelty of this work? The author should add more lines related to it in the introduction section or below the abstract.

Please mention properly about the procedures of recyclability of the adsorbents used in this study as it reflects the sustainability and cost-effectiveness of the adsorbents e.g. see”. Please explain. (Cite these papers as an example.) <https://link.springer.com/article/10.1007/s13369-022-07015-w>, https://www.sciencedirect.com/science/article/pii/S0927775721017489.
