# Supplementary material for: The study on the adsorption characteristics of anthracite under different temperature and pressure conditions
Source: PLoS One. 2025 Mar 11;20(3):e0310863. doi: 10.1371/journal.pone.0310863 (PMC11896062; doi:10.1371/journal.pone.0310863)
Supplement: Attachment — Submitted filename: Response to Reviewers .docx [file pone.0310863.s004.docx]

Dear editor:

Thank you very much for giving us an opportunity to revise our manuscript. We appreciate the editor and reviewers very much for their constructive comments and suggestions on our manuscript entitled“**The study on the adsorption characteristics of anthracite under different temperature and pressure conditions**”(ID: PONE-D-24-04323).

We have studied reviewers' comments carefully. According to the reviewers' detailed suggestions. we have made a careful revision on the original manuscript. I modified the copy of the markup to form a separate file, named 'Revised Manuscript with Track Changes'.

Kind regards.

Corresponding author: Dan Zhao

E-mail address: 2373562966@com

Replies to the reviewers’ comments:

Reviewer #1:

1. In the introduction section what does the Author mean by “stress condition” Please explain.

Response: I am very sorry for your misunderstanding due to my unclear expression. "stress condition" in the article refers to the triaxial stress exerted on coal pillar during the experiment, and "stress condition" can also be understood as a set of variables set in the experiment.

2. I suggest to further elaborate more about point of zero charge analysis to know the surface charge and its effect should be included in the methodology and results section. The author can refer these articles https://www.tandfonline.com/doi/full/10.1080/07391102.2023.2186704.

Response: Thanks for the reviewer's suggestions, I have read this article closely and found that this article describes the surface charge and its influence law in detail, which has a strong guidance and reference significance for the molecular simulation part of this paper, so I cite this article, which is reflected as follows:

[34]Çetinkaya Serap,Eyupoglu Volkan,Çetintaş Halil İbrahim, et al. Removal of Erythrosine B dye from wastewater by Penicillium italicum: experimental, DFT, and molecular docking studies.[J].Journal of biomolecular structure dynamics,2023,41(23):11-12. doi: 10.1080/07391102.2023.2186704.

3. What is the novelty of this work? The author should add more lines related to it in the introduction section or below the abstract.

Response: Thanks for the reviewer's suggestions on innovation points. Due to the problems I expressed, the innovation points in this paper are too scattered and there is no clear condensed innovation points. The innovation of this paper is mainly divided into two points. The first innovation is that the previous authors mostly used pulverized coal for experimental research. In this paper, cylindrical raw coal was used for experiments, and based on the coal-rock adsorption test platform under multi-physical field conditions, the isothermal adsorption experiment of CO_2_/CH_4_/N_2_ by cylindrical anthracite was carried out. The experimental arrangement is closer to the working condition of the coal seam, and the law of the change of adsorption capacity with gas injection pressure is obtained. The second innovation is the establishment of the CH_4_/CO_2_/N_2_ adsorption conformation model of single molecule structure of anthracite at different temperatures, and the pore information obtained by different gases as probes is different, and the simulation results can supplement the deficiency that the experiment cannot observe the microscopic changes. Based on this, I made supplementary revisions in the introduction part of the manuscript, as follows:

Their results revealed that the model can predict the adsorption behavior of CH_4_, N_2_, and CO_2_ on coal and considers the influence of adsorption surface structure characteristics on the adsorption behavior, which can improve the ability to predict high-pressure adsorption phenomenon. Through the above analysis, we can see that at present, some research has been conducted ^[22–24]^. However, there are fewer experiments that use columnar coal for analysis, and there is relatively little research on the adsorption micro-mechanism using molecular simulation combined with experiments.

Given this, in this paper, a coal rock adsorption test platform under multi-physical field conditions is applied to explore the variations in and controlling mechanism of the coal adsorption capacity with adsorption pressure during the adsorption of CO_2_, CH_4_, and N_2_ by columnar anthracite from the Yangquan Coal Mine, Shanxi Province, China, under stress conditions. Based on experimental research and through molecular dynamics simulation, the pore structure characteristics of an anthracite macromolecular structure model and the influence of temperature on the adsorption performance of the anthracite molecular structure model for single component CO_2_, CH_4_, and N_2_ gases were studied from the microscopic point of view. The research findings presented in this paper are valuable for enhancing our understanding of the adsorption characteristics and mechanisms of CH_4_, CO_2_, and N_2_ in coal. The aim is to elucidate the influence of factors such as adsorption pressure, temperature, gas properties, and other variables on the molecular-level adsorption and diffusion behaviors of gases. This study provides theoretical support and technical guidance for optimizing CH_4_ treatment and CO_2_ geological storage in coal mines.

4. Please mention properly about the procedures of recyclability of the adsorbents used in this study as it reflects the sustainability and cost-effectiveness of the adsorbents e.g. see”. Please explain. (Cite these papers as an example.) https://link.springer.com/article/10.1007/s13369-022-07015-w, <https://www.sciencedirect.com/science/article/pii/S0927775721017489>.

Response: Thanks for the reviewer's suggestions, I read this article closely and found that these two articles described the recyclability procedure of adsorbent in detail, reflecting the sustainability and cost-effectiveness of adsorbent, which provided strong guidance and reference significance for the research on coal adsorption in this paper. Therefore, these two articles were quoted and reflected in the introduction of the article as follows:

[13]Tauqir A ,Saood M M ,Ullah S K , et al.Synthesis and Adsorptive Performance of a Novel Triazine Core-Containing Resin for the Ultrahigh Removal of Malachite Green from Water[J].Arabian Journal for Science and Engineering,2022,48(7):8571-8584. doi：10.1007/S13369-022-07015-W.

[15]Dalhat N M ,Saood M M ,Mukarram Z , et al.Volcanic ashe and its NaOH modified adsorbent for superb cationic dye uptake from water: Statistical evaluation, optimization, and mechanistic studies[J].Colloids and Surfaces A: Physicochemical and Engineering Aspects,2022,634. doi：10.1016/J.COLSURFA.2021.127879.

Thanks to the reviewer for carefully reading my manuscript. According to your suggestions, I read my manuscript again and corrected some language errors. Your comments on the review of this paper have improved the academic level of this paper as a whole, and I would like to express my thanks to you again!

Reviewer #2:

1. The figure is somewhat blurred, it is recommended to replace the high-definition figure. 1.

Response: Thanks for the reviewer's careful review of my manuscript. Due to my negligence, some numerical simulation images were not clear, so I revised and uploaded the high-definition figures again. The revised figures are as follows:

(a) Three-dimensional model of anthracite	(b) Crystal cell model

Fig.2 Numerical model of anthracite

(a) CO_2_ gas	(b) CH_4_ gas

(c) N_2_ gas

Fig.4 Variation in the gas adsorbed by the coal samples under effective stress

Fig. 6 Position of each slice of anthracite

2. The introduction needs to describe in more detail the background of the study, why anthracite coal was chosen as the subject of the study, and the specific implications of this study for coalbed methane control and CO2 geological storage. Some of the references have little to do with the research content of this paper, and it is suggested that appropriate deletions be made or more closely related articles be added.

Response: Thanks to the reviewer for pointing out the shortcomings of the introduction part of this paper. I fully agree with your point of view and greatly enhance the theoretical value of this paper through your guidance. First, I added the main reasons for choosing anthracite as my research topic in the introduction, and explained the specific impact of this research on coal-bed methane control and CO_2_ geological storage. Some less relevant references are deleted, and more closely related references about coal bed methane are added. Specific modifications are as follows:

As the main energy source in China, coal has provided strong support for social and economic development and will continue in its role as a ballast stone in maintaining stable economic operations for a long time in the future ^[1,2]^. China's anthracite resources are very rich, accounting for 14% of the country's total coal reserves, while anthracite contains a large number of coal-bed methane resources, but coal mine gas disasters still occur from time to time ^[3,4]^. The main reason for their occurrence is that the research on the development theory and technology of coalbed methane (CH_4_, CO_2_, N_2_, and other gases) is relatively weak. In particular, the understanding of the adsorption mechanism of coalbed methane is still insufficient, resulting in the unsatisfactory development effect of coalbed methane. Therefore, it is urgent to study the adsorption characteristics of coalbed methane in a reservoir ^[5]^

Given this, in this paper, a coal rock adsorption test platform under multi-physical field conditions is applied to explore the variations in and controlling mechanism of the coal adsorption capacity with adsorption pressure during the adsorption of CO_2_, CH_4_, and N_2_ by columnar anthracite from the Yangquan Coal Mine, Shanxi Province, China, under stress conditions. Based on experimental research and through molecular dynamics simulation, the pore structure characteristics of an anthracite macromolecular structure model and the influence of temperature on the adsorption performance of the anthracite molecular structure model for single component CO_2_, CH_4_, and N_2_ gases were studied from the microscopic point of view. The research findings presented in this paper are valuable for enhancing our understanding of the adsorption characteristics and mechanisms of CH_4_, CO_2_, and N_2_ in coal. The aim is to elucidate the influence of factors such as adsorption pressure, temperature, gas properties, and other variables on the molecular-level adsorption and diffusion behaviors of gases. This study provides theoretical support and technical guidance for optimizing CH_4_ treatment and CO_2_ geological storage in coal mines.

References [9] and [17] in the original manuscript have been deleted, and new references related to coal bed methane have been added, as follows:

Ranathunga^[9]^ studied the applicability of CO_2_ to enhance coalbed methane recovery through CO_2_-driven coalbed methane experiment, and the results showed that CO_2_ injection had a higher coalbed methane recovery rate than natural extraction, and effectively improved the anti-reflection performance of coal seams.

Zeng^[17]^ simulated the coal-bed methane mining process using the adsorption-strain coupling model, and the results showed that the microsimulation could explore the changes in coal deformation and permeability that were difficult to find in experiments.

3. Much has been done by previous authors to address the effect of temperature and pressure on the adsorption characteristics of anthracite. How to show the innovation of this paper?

Response: Thanks for the reviewer's suggestions on innovation points. Due to the problems I expressed, the innovation points in this paper are too scattered and there is no clear condensed innovation points. The innovation of this paper is mainly divided into two points. The first innovation is that the previous authors mostly used pulverized coal for experimental research. In this paper, cylindrical raw coal was used for experiments, and based on the coal-rock adsorption test platform under multi-physical field conditions, the isothermal adsorption experiment of CO_2_/CH_4_/N_2_ by cylindrical anthracite was carried out. The experimental arrangement is closer to the working condition of the coal seam, and the law of the change of adsorption capacity with gas injection pressure is obtained. The second innovation is the establishment of the CH_4_/CO_2_/N_2_ adsorption conformation model of single molecule structure of anthracite at different temperatures, and the pore information obtained by different gases as probes is different, and the simulation results can supplement the deficiency that the experiment cannot observe the microscopic changes.

4. Was the model validated for reasonableness?

Response: Thanks for the reviewer's questions about the verification of the model's rationality. I strongly agree with the reviewer's suggestions, because the rationality of the model structure directly affects the accuracy of the simulation results, so I am very sorry for causing your misunderstanding due to the problems I expressed. Since the model I cited was self-constructed and the model construction process and rationality verification have been published in the early edition of Plos one, I did not give too much description. After referring to your opinions, I added some general descriptions about this part in the paper, and your suggestions made the expression of this paper more clear.

The references for the anthracite model I independently constructed are as follows:

[26]Jia Jinzhang,Wu Yumo,Zhao Dan, et al. Molecular structure characterization analysis and molecular model construction of anthracite.[J]. PloS one,2022,17(9). doi:10.1371/JOURNAL.PONE.0275108.

In the article on the construction of anthracite model, the expression on the rationality verification of the model is as follows:

4.2 The anthracite coal molecular model validation

To further verify the rationality of the parameters of the molecular model of anthracite, the molecular structure cell model of anthracite was established, and the adsorption of CH_4_ gas molecules in the molecular structure model of anthracite was observed. The molecular structure models of 15 anthracites were obtained, and the Amorphous Cell module was used. The calculation accuracy was fine, and we used the COMPASS force field. The 15 single molecular structures were placed into the cell to add three-dimensional periodic boundary conditions, and the density was set to 1.32 g/cm^3^. Then, structure optimization and dynamic optimization of the cell model of anthracite were carried out to minimize and stabilize the energy of the constructed coal molecular structure model. Finally, the structure model size of anthracite with low energy conformation was A = B = C = 3.89034 nm, where the molecular formula was C_3120_H_2430_N_60_O_180_. The cell model of the anthracite macromolecular structure is shown in Fig. 12.

Fig 12 Cellular model of the molecular structure of anthracite

MS software was used to analyze the adsorption of CH_4_ gas molecules in the molecular structure model of anthracite by combining GCMC and MD. The simulation process was completed by the adsorption and Forcite modules in MS. The adsorption data of the CH_4_ molecules in the anthracite molecular model were compared with the experimental data of anthracite in reference 17, as shown in Fig. 13. The comparison showed that the adsorption amount of CH_4_ gas was within an order of magnitude, which proved that the anthracite molecular model established in this work conformed to the structural characteristics of anthracite. However, the columnar coal samples used in the adsorption experiment had defects to some extent, resulting in a decrease in the pore volume and specific surface area in the coal body, which reduced the gas adsorption capacity in the experimental process. The variation trend of the CH_4_ adsorption amount obtained by the experiments and molecular simulation was basically consistent, which further proved that the anthracite molecular model established in this work was reasonable.

Fig 13 Comparison of CH_4_ adsorption simulation and experiment

5. The description of the molecular simulation section is too simple and needs to be supplemented with some information on simulation steps, parameter settings, and so on.

Response: Thanks to the reviewer for pointing out the shortcomings of the description of simulation Settings. I have supplemented and modified the questions you raised, and the modified results are as follows:

The adsorption mechanism of the single component CO_2_, CH_4_, and N_2_ gas molecules on the anthracite macromolecular structure model was analyzed using the grand-canonical Monte Carlo and molecular dynamics methods. The gas adsorption simulation was performed using the fixed pressure task in the sorption module. Set the radius of Connolly probe to 0.13nm (molecular dynamics radius of He), the mesh resolution to 0.75 Å, Equilibration steps and Production steps to 1000000. The COMPASS force field was selected (this force field is suitable for simulating the adsorption of small gas molecules such as CO2 and CH4 in coal macromolecules).

6. Section 3.2 Second paragraph units note superscript.

Response: Thanks to the reviewer for finding this problem in my manuscript. I am very sorry that such low-level mistakes occurred due to my negligence. I have revised this part and guaranteed that such problems will not occur in the future, and the modified results are as follows:.

In the macromolecular structure of anthracite, the micropore volume detected for CO_2_ was 2975.78 Å^3^, and the specific surface area was 3787.95 Å^2^. The micropore volume detected for CH_4_ was 2118.40 Å^3^, and the specific surface area was 2705.77 Å^2^. The micropore volume detected for N_2_ was 2327.42 Å^3^, and the specific surface area was 3021.81 Å^2^. In the macromolecular structure of anthracite, the micropore volume and specific surface area detected for the CO_2_ molecules were significantly higher than those for CH_4_, followed by those for N_2._ This explains why some micropores in the coal body can be detected using CO_2_ molecules but not using CH_4_ and N_2_ molecules. Similarly, for the same micropore, the space detected using CO_2_ molecules is larger than that detected using CH_4_ and N_2_ molecules. In terms of the adsorption space provided by the micropores in the coal body, the CO_2_ gas was more easily adsorbed by the coal body than the CH_4_ and N_2_ gas.

7. The conclusion section is too brief and it is recommended to summarize the main innovations, findings and implications of the article and point out directions for future work.

Response: Thanks for the reviewer's guidance on the conclusion of this paper, which enabled me to find the shortcomings of the conclusion. Therefore, according to your suggestions, I summarized the innovation points, findings and Revelations of this paper in the conclusion, and pointed out the future direction of work, and the modified results are as follows:

In this study, the characteristics of CO_2_, CH_4_, and N_2_ adsorption on columnar coal were studied using an experimental platform for coal rock gas adsorption tests under multi-physical field conditions. Compared with the use of pulverized coal as the research object, the experimental arrangement is closer to the working condition of coal seam, which improves the accuracy of the conclusion. Moreover, the molecular simulation method is used to produce differences in the pore information obtained by different gases as the probe, which supports the experimental results. The simulation results can supplement the shortcomings of the experiment in observing microscopic changes. The main conclusions of this study are as follows.

(1) The gas adsorption capacities of anthracite are CO_2_ > CH_4_ > N_2_, and the adsorption capacity increases with increasing gas injection pressure. When the pressure is less than 3 MPa, the adsorption capacities of anthracite for the three gases increase relatively quickly, and the isothermal adsorption curves are steep. When the pressure is greater than 3 MPa, the adsorption capacities of anthracite for CH_4_ and N_2_ increase relatively slowly, and the adsorption capacities tend to become saturated, while the CO_2_ adsorption capacity exhibits a steep increase at approximately 4 MPa.

(2) The distribution characteristics of the micropores and pores in the macromolecular structure model of anthracite were revealed using the probe method. The smaller the kinetic diameter of gas molecules is, the more pores the gas can enter, and the larger the pore surface area that can come in contact with is. There are both accessible and inaccessible micropores.

(3) The CO_2_, CH_4_, and N_2_ adsorption capacities of the anthracite model increased with increasing adsorption pressure, while the gas adsorption increment of the coal exhibited the opposite trend, which is consistent with the law obtained from the experiments.

(4) The CO_2_, CH_4_, and N_2_ adsorption conformation models of the anthracite monomolecular structure at different temperatures were established. An increase in temperature was not conducive to the adsorption of gas molecules by the coal macromolecular model, and the increase in temperature had the greatest influence on the adsorption capacity of CO_2_, followed by those of CH_4_ and N_2_. The research results lay a theoretical foundation for the molecular study of gas adsorption behavior by coal molecular model. It provides technical support for the follow-up study of coalbed methane Wells to formulate reasonable drainage and production methods

8. Harmonize reference formats as required.

Response: Thanks to the reviewer's suggestions, I modified the template according to Plos One journal. Finally, thanks to the reviewer for his valuable suggestions during his busy schedule. Based on your guidance, the quality of this paper has been significantly improved.
