# Supplementary material for: The study on the adsorption characteristics of anthracite under different temperature and pressure conditions
Source: PLoS One. 2025 Mar 11;20(3):e0310863. doi: 10.1371/journal.pone.0310863 (PMC11896062; doi:10.1371/journal.pone.0310863)
Supplement: Attachment — Submitted filename: Response_to_Reviewers__auresp_2.docx [file pone.0310863.s006.docx]

Dear editor:

Thank you very much for giving us an opportunity to revise our manuscript. We appreciate the editor and reviewers very much for their constructive comments and suggestions on our manuscript entitled“**The study on the adsorption characteristics of anthracite under different temperature and pressure conditions**”(ID: PONE-D-24-04323).

We have studied reviewers' comments carefully. According to the reviewers' detailed suggestions. we have made a careful revision on the original manuscript. I modified the copy of the markup to form a separate file, named 'Revised Manuscript with Track Changes'.

Kind regards.

Corresponding author: Dan Zhao

E-mail address: 2373562966@com

Replies to the reviewers’ comments:

Comments:

1. Both the abstract and conclusion parts need to show the innovation of this paper to differentiate the present work from others.

Response: Thank the reviewers for their suggestions on the abstract part of the manuscript. In order to highlight the innovation of this article, I revised the abstract as follows :

The study of the adsorption characteristics of coal is of great significance to gas prevention and CO_2_ geological storage. To explore the adsorption mechanism of coal, this study focuses on columnar anthracite. Adsorption tests on coal rock under a range of physical field conditions were conducted using the volumetric method. The adsorption characteristics of anthracite for CO₂, CH₄, and N₂ gases under different conditions were investigated using Grand Canonical Monte Carlo (GCMC) and Molecular Dynamics (MD) methods. The results showed that the adsorption capacities of anthracite for these three gases are in the order of CO_2_ > CH_4_ > N_2_, and that the adsorption capacity increases with increasing gas injection pressure. The CO_2_/CH_4_/N_2_ gas molecule adsorption capacity of the anthracite macromolecular structure model decreases with increasing temperature. The increase in temperature has the greatest influence on the CO_2_ absorption capacity, followed by the CH_4_ and N_2_ adsorption capacities. The research offers a theoretical basis for the control of coal mine gas and the geological storage of CO_2_.

2. What was the main reason for selecting the anthracite coal for this research? It should be mentioned carefully in detail at the beginning of the Introduction section.

Response: I would like to thank the reviewer for identifying the deficiencies in my Introduction section. I am deeply sorry that, due to my negligence, the primary reason for selecting anthracite as the subject of the experiment was not adequately emphasised. In light of the above, I have made the following revisions to the manuscript:

As a crucial raw material for deep coal processing, anthracite has been instrumental in the early stages of China's coal chemical industry. Concurrently, a considerable volume of coalbed methane resources is present within anthracite, yet incidents of coal mine gas disasters persist. The principal reason for this is that research into the development theory and technology of coalbed methane (CH_4_, CO_2_, N_2_ and other gases) is relatively limited. In particular, there is still a lack of understanding of the mechanism of adsorption characteristics of coalbed methane, which is resulting in an unsatisfactory development effect of coalbed methane. Accordingly, this study employs anthracite as the experimental coal sample to investigate the adsorption characteristics of coalbed methane in the reservoir, which represents a critical and pressing issue in the development and research of coalbed methane.

3. In section 3.1., it was mentioned that “The larger the polarizability of the gas molecule is, the greater the dispersion force and induction force are. This makes it easier for the gas to occupy the adsorption site on the surface of the”. It means that the higher polarizability of CO_2_ molecules facilitates their adsorption on the adsorbent’s surface compared to other gases. Therefore, it is recommended that the Authors work on the schematic diagram of interactions between anthracite and the desired molecule (i.e., CO_2_) for more clarification.

Response: Thank you for the reviewer 's conclusion that CO_2_ is more adsorbable than CH_4_ and N_2_ in a schematic way. I strongly agree with your opinion. I used molecular dynamics to simulate the adsorption configuration of three gases in seven main functional groups in coal. The conclusion is consistent with the experimental results, but this simulation study I and our team 's Professor Jinzhang J have published in the Fuel journal in 2023 ( Jinzhang J,Yumo W ,Dan Z , et al. Adsorption of CH_4_/CO_2_/N_2_ by different functional groups in coal[J].Fuel,2023,335 ), so this sentence I want to express is explained in the form of a conclusion. In this regard, I have added a reference to the article to illustrate this problem. The adsorption configurations of seven main functional groups of three gases in coal are as follows:

Stable configurations of CO_2_adsorbed by fragments of different functional groups

Stable configurations of CH_4_ adsorbed by fragments of different functional groups

Stable configurations of N_2_ adsorbed by fragments of different functional groups

4. What is the meaning of the “effective stress”? It has been mentioned several times in section 3.1 without any clarification. Please define it before and explain clearly how this factor(s) impacts the gas adsorption capacity of the anthracite used.

Response: I would like to thank the reviewer for the explanation of ‘effective stress’, and I am very sorry that I did not write down the origin of ‘effective stress’ in the paper due to my negligence. The ‘effective stress’ is the stress applied to the coal samples by the experimental test rig in this paper, which is based on the applied axial and peripheral pressures to simulate the depth of burial, reservoir pressure, temperature and geostress distribution of the coal samples. Therefore, I have added a description of the ‘effective stress’ setting in Summary 2.1 of the manuscript, as shown below:

In this experiment, the experimental test of adsorption of pure CH_4_, CO_2_ and N_2_ by anthracite coal was carried out by the isovolumetric method.According to the burial depth of the experimental coal samples, the reservoir pressure, the temperature and the distribution of the geostresses, the final setup of the experimental temperature was 30°C. The axial pressure of the test samples was applied at 10 MPa, the enclosing pressure was applied at 8 MPa, and the equilibrium pressure of the adsorption was taken as 7 points, ranging from 0.5 to 6 MPa.For the convenience of the calculations, the effective stress is defined as: 1 / 3 × [ Axial pressure + 2 × Confining pressure ] -Equilibrium pressure.

5. The language of this paper needs to be improved at the academic level as a whole. Active or passive sentences were not carefully described. The Authors must correct the language errors. Some are:

i. Abstract “The results show that the adsorption capacities of anthracite for these three gases are”. The results showed that...
ii. Section 1 Introduction “He gas had a weak adsorption capacity with an almost negligible volume change”. He is a specific gas. Use an article before that “The He”. It has been repeated many times in the drafts, please correct all.
iii. Section 1 Introduction “However, there are fewer experiments that use columnar coal for analysis, and there is relatively little research on the adsorption micro-mechanism using molecular simulation combined with experiments”. There are fewer experiments that should be changed to “fewer experiments use”.
iv. Section 2.1 “F8 were opened to vacuum the system to discharge any impurities such as air and water from the enture device”. What is the meaning of enture? It might be entire.
v. Section 2.1 “After the free volume test was completed, the system was vacuumized again to ensure that He gas inside the system and the coal sample was completely pumped out to avoid experimental errors”. There is no vocabulary in English for vacuumized. Vacuumed is the correct form.
vi. Section 3.2 “The smaller the radius of the probe molecule is, the larger the pore volume that can be detected is”. The second is should be deleted.
vii. Section 3.3 “To further explore the pore distribution characteristics of the anthracite macromolecular structure, on the basis of obtaining the pore distribution characteristics”. On the basis of should be changed to based on.

Response: Thank you for reading my manuscript carefully. I apologize for the numerous grammatical issues found in the manuscript and greatly appreciate your patience in helping me correct them. I have revised the entire text according to your suggested changes and have reviewed and amended the manuscript thoroughly using your method.

Reviewer #3: The authors have improved the manuscript, and most of the comments have been taken into consideration. Consequently, I suggest publishing the paper after minor revision. It is worth to be noted that the unit of specific surface area and total pore volume are Å2/g and Å3/g, respectively. Please correct these units in the manuscript.

Response: I would like to thank the reviewers for their valuable comments on my manuscript and their recognition of its quality. I apologize for any mistakes that were made due to my negligence, and I have made the necessary changes to the manuscript according to your suggestions.

Reviewer #4: This work presented a study on the potential of anthracite coal as an effective medium for adsorbing CO_2_. Nonetheless, the language of this paper needs to be improved at the academic level as a whole. Active or passive sentences were not carefully described. The Authors must correct the language errors

Response:I would like to thank the reviewers for their valuable comments on my manuscript and their recognition of its quality. I apologize for the grammatical errors in the manuscript due to my oversight. I have reviewed and revised the entire manuscript according to your suggestions.

Reviewer #5: The manuscript titled "The study on the adsorption characteristics of anthracite under different temperature and pressure conditions" offers valuable insights into how anthracite interacts with CO₂, CH₄, and N₂ under various temperature and pressure scenarios. Given the increasing focus on carbon sequestration and methane recovery, this research is both timely and relevant. However, after carefully reviewing the manuscript, I believe it needs significant revisions before it can be considered for publication. Below, I’ve outlined the main concerns and recommendations that should be addressed in the revised version.

Abstract:

1. The abstract effectively summarizes the key findings regarding the adsorption capacities of CO₂, CH₄, and N₂ on anthracite under varying conditions. However, it lacks a discussion on the practical implications of these findings. How do these results inform the potential for industrial applications, particularly in CO₂ sequestration or CH₄ recovery?

2. The abstract should also mention the methodology, particularly the use of molecular simulation alongside experimental approaches, as this is a significant aspect of the study.

3. Consider adding a brief mention of the study's limitations, such as the reliance on a specific coal type (anthracite) and how this might affect the generalizability of the findings.

4. The paragraph uses a repetitive sentence structure, with most sentences beginning in a similar way ("The study of...," "To explore...," "The results show..."). This can make the text monotonous and harder to engage with. Varying sentence structures would make the text more dynamic and easier to read.

5. The text could be more concise. Phrases like "in this paper, columnar anthracite is taken as the research object" could be simplified to "this study focuses on columnar anthracite.

Response: Thanks to the reviewer 's revision of the abstract part of the manuscript, I learned a novel way of writing. I think that modifying the abstract according to your requirements can make the writing focus of this article clearer and easier to understand, so that this article has been raised to a higher level. The abstracts modified according to the several amendments you proposed above are as follows :

The study of the adsorption characteristics of coal is of great significance to gas prevention and CO_2_ geological storage. To explore the adsorption mechanism of coal, this study focuses on columnar anthracite. Adsorption tests on coal rock under a range of physical field conditions were conducted using the volumetric method. The adsorption characteristics of anthracite for CO₂, CH₄, and N₂ gases under different conditions were investigated using Grand Canonical Monte Carlo (GCMC) and Molecular Dynamics (MD) methods,The results showed that the adsorption capacities of anthracite for these three gases are in the order of CO_2_ > CH_4_ > N_2_, and that the adsorption capacity increases with increasing gas injection pressure. The CO_2_/CH_4_/N_2_ gas molecule adsorption capacity of the anthracite macromolecular structure model decreases with increasing temperature. The increase in temperature has the greatest influence on the CO_2_ absorption capacity, followed by the CH_4_ and N_2_ adsorption capacities. The research offers a theoretical basis for the control of coal mine gas and the geological storage of CO_2_.

Introduction:
1. The introduction provides a thorough background on the significance of studying adsorption characteristics in coal, particularly anthracite. However, it could be condensed to improve readability and focus more directly on the study's objectives.

Response: Thank you for the guidance provided by the reviewers, and I agree with you very much. Therefore, I deleted two redundant references to make the background description of the adsorption characteristics of anthracite more concise.

2. While the introduction references prior studies, it could benefit from a more detailed comparison with previous research, especially in terms of how this study advances our understanding of the topic. Specifically, what gaps in the literature does this study aim to fill? To strengthen this section, I recommend citing recent studies that have explored similar topics in the context of carbon capture and storage (CCS) and methane recovery, such as those by https://doi.org/10.1016/j.envres.2023.116879 and https://doi.org/10.1016/j.jece.2023.110833. These articles provide relevant insights and comparisons that can help contextualize the novelty of your work, particularly in how your study contributes to the ongoing development of efficient adsorption technologies. Citing these sources will also demonstrate how your research builds upon and extends the current state of knowledge in this critical area.

Response: Thank the reviewers for helping me find two excellent references. I think these two articles have profound insights in the field of studying how to apply efficient technology to improve adsorption. After intensive reading of the two articles, I benefited a lot. Therefore, I added these two papers as references in my manuscript, as shown below :

[9] Ul W M ,Ul M S H ,Nasir S S , et al. Enhancing CO_2_ separation from N_2_ mixtures using hydrophobic porous supports immobilized with tributyl-tetradecyl-phosphonium chloride [P44414][Cl].[J].Environmental research,2023,237(P1):116879-116879. doi：10.1016/j.envres.2023.116879

[13] Tauqir A ,Saood M M ,Ullah S K , et al.Synthesis and Adsorptive Performance of a Novel Triazine Core-Containing Resin for the Ultrahigh Removal of Malachite Green from Water[J].Arabian Journal for Science and Engineering,2022,48(7):8571-8584. doi：10.1007/S13369-022-07015-W.

3. The introduction should highlight the novelty of the study more clearly. What distinguishes this study from previous work on similar topics? Is it the combination of experimental and simulation approaches, the specific conditions tested, or the focus on anthracite?

Response: As evidenced by the preceding analysis, research has been conducted on the adsorption characteristics of coal seams. Nevertheless, the majority of scholars have focused their research on pulverised coal, with relatively few studies examining columnar coal, which is in closer proximity to the coal storage environment. Additionally, there is a paucity of research examining the microscopic mechanisms of adsorption through the integration of molecular simulation and experimentation.

Given this, in this paper, a coal rock adsorption test platform under multi-physical field conditions is applied to explore the variations in and controlling mechanism of the coal adsorption capacity with adsorption pressure during the adsorption of CO_2_, CH_4_, and N_2_ by columnar anthracite from the Yangquan Coal Mine, Shanxi Province, China, under stress conditions. Based on experimental research and through molecular dynamics simulation, the pore structure characteristics of an anthracite macromolecular structure model and the influence of temperature on the adsorption performance of the anthracite molecular structure model for single component CO_2_, CH_4_, and N_2_ gases were studied from the microscopic point of view. The research findings presented in this paper are valuable for enhancing our understanding of the adsorption characteristics and mechanisms of CH_4_, CO_2_, and N_2_ in coal. The aim is to elucidate the influence of factors such as adsorption pressure, temperature, gas properties, and other variables on the molecular-level adsorption and diffusion behaviors of gases. This study provides theoretical support and technical guidance for optimizing CH_4_ treatment and CO_2_ geological storage in coal mines.

4. While the text does a thorough job of summarizing past studies, it does not sufficiently highlight how this study contributes something new to the field. The discussion of previous research dominates the introduction, but the authors do not clearly articulate how their work differs from or builds upon these studies.

Response: Thanks for the reviewer 's guidance, I very much agree with you. In order to clarify the difference between the previous research work and these current methods in the introduction part, and to explain how the work is based on these studies, this part is added in the introduction part. The description is to enhance the cohesion of the paragraph, as shown below :

A substantial body of research has been conducted by scholars in the field of coal adsorption capacity. However, the constraints imposed by experimental conditions have resulted in the inability to derive more than macroscopic laws from the observed phenomena. Many factors affect the adsorption characteristics of coal, and the microscopic adsorption mechanism cannot be fully revealed through experimental methods. Therefore, many scholars have used molecular simulation methods and experiments to research these topics, which can make up for the defects of experiments and can effectively reveal the influence mechanism of the gas adsorption performance.

Materials and Methods:
1. The methodology is well-detailed, particularly the description of the experimental setup and the conditions under which the adsorption tests were conducted. However, there is limited discussion on the reproducibility of the experiments. How were variations in the coal samples (e.g., porosity, moisture content) controlled or accounted for?
 Response: Thanking the reviewers for their suggestions, I added this part of the description to the manuscript, as follows :

When the adsorption of each target pressure coal sample reached equilibrium, the system automatically calculated the amount of adsorption as follows:

$Q_{i}=\frac{V_{m}\left(\frac{P_{i0}V_{us}}{Z_{i0}}-\frac{P_{i1}\left(V_{us}+V_{f}\right)}{Z_{i1}}\right)}{mRT}$, (1)

where *V_m_* is the molar volume of the gas (22.4 L/mol); *P_i0_* is the initial pressure of the ith upstream reference tank (MPa); *V_us_* is the system automatic test upstream reference tank and pipeline volume; and *V_f_* is the volume of the free space. Additionally, *Z_i0_* is the initial compression factor of the ith gas, *P_i1_* is the equilibrium pressure of the gas in the ith reference tank (MPa); *Z_i1_* is the compression factor of the ith equilibrium gas; R is the gas molar constant; and T is the experimental temperature (K).

2. The rationale behind the selection of the specific temperature and pressure ranges should be explained. How do these conditions relate to real-world scenarios in coal mining or carbon sequestration projects?

Response: The temperature and pressure set by the experiment are corresponding to the temperature and pressure of the coal sample in the coal mine. Thanks to the reviewer 's suggestion, I realized that the manuscript 's description of these aspects is insufficient. I added this part of the description in the manuscript, as shown below :

In this experiment, the experimental test of adsorption of pure CH_4_, CO_2_ and N_2_ by anthracite coal was carried out by the isovolumetric method.According to the burial depth of the experimental coal samples, the reservoir pressure, the temperature and the distribution of the geostresses, the final setup of the experimental temperature was 30°C. The axial pressure of the test samples was applied at 10 MPa, the enclosing pressure was applied at 8 MPa, and the equilibrium pressure of the adsorption was taken as 7 points, ranging from 0.5 to 6 MPa.For the convenience of the calculations, the effective stress is defined as: 1 / 3 × [ Axial pressure + 2 × Confining pressure ] -Equilibrium pressure.

3. The molecular simulation method is described, but the choice of parameters (e.g., force field, probe radius) should be justified. Why were these particular settings chosen, and how do they affect the simulation outcomes?

Response: Thanks to the reviewer 's professional suggestions, first of all, in the selection of force field, we found that the coal sample selected in this paper has strong stability in the range of 0-6MP.After exceeding 6MP, the coal structure is prone to large deformation, which affects the experimental results. Secondly, in terms of probe radius, I put the parameter settings in Section 3.2 to explain : The pore volume and specific surface area of the coal macromolecular structure are not fixed, and the pore characteristics are closely related to the molecular radius of the Connolly probe ^[33,34]^. The smaller the radius of the probe molecule is, the larger the pore volume that can be detected is. The gases used in this study were CO_2_, CH_4_, and N_2_, and the probe radius was equivalent to the molecular dynamics radii of the three gases, namely, 0.165 nm, 0.19 nm, and 0.182 nm.

Results and Discussion:
1. The results are presented clearly, with appropriate use of figures and tables to illustrate key findings. However, the discussion often remains descriptive, with limited interpretation of the results. For example, the observed trends in adsorption with varying pressure and temperature could be linked more explicitly to the molecular interactions at play.

Response: Thank you to the reviewers for reading my manuscript carefully. I agree with you very much. I am very sorry, I have some deficiencies in the description of the experiment, but according to your review opinion, I have learned a lot. Therefore, I have made some modifications in the text, trying to strengthen the explanation of the conclusion. At the same time, I will also pay attention to the strengthening of writing techniques in future research.

2. The study uses the Langmuir model to fit the adsorption data, but the choice of this model over others (e.g., Freundlich, Temkin) is not justified. A discussion on the model's applicability to the experimental data, including any limitations or deviations, would strengthen the analysis.

Response: Thanks to the reviewer 's attention to the analytical methods in the manuscript, I found that the Langmuir model is suitable for monolayer adsorption and homogeneous surfaces when selecting analytical methods. The Freundlich model is suitable for multi-molecular layer adsorption and non-uniform surface. The Temkin model is suitable for considering the finite interaction between adsorbates and the linear distribution of adsorption heat. For the coal molecules in this paper, it is more inclined to monolayer adsorption and uniform surface. At the same time, many scholars in the industry mostly choose Langmuir model in the study of coal adsorption, and have achieved more results. Therefore, this paper also chooses Langmuir model for analysis, and the results are in good agreement with the experiment.

3. The discussion on the impact of temperature on adsorption is thorough but could benefit from a comparison with similar studies. How do the findings compare with previous research on different coal types or adsorption conditions?

Response: Thank the reviewers for their concern about whether the conclusions in the manuscript are consistent with the results of other scholars. In this paper, the method of different gas adsorption conformation models is used to analyze the results of temperature on adsorption. Although the method is innovative, the conclusions obtained are in line with the basic principles and consistent with previous studies. In the selection of coal samples, this paper chooses the molecular structure model of anthracite for related research. The molecular structure model of different coal samples only has a certain gap in the number of atoms and functional groups. Although the adsorption capacity of different coal samples will be different, the overall law is similar.

4. The molecular simulation results are interesting but could be integrated more closely with the experimental findings. For instance, how do the pore structures observed in the simulations correspond to the experimental adsorption data?

Response: Thanks to the reviewer 's question about the correlation between the experiment and the simulation results, I also think this is very critical, so I describe it in Section 3.2 of the article :

Through analysis of the pore distribution characteristics of the anthracite macromolecular model, the characteristics of the micropore morphology, distribution, connectivity, and heterogeneity of the anthracite macromolecular structure were revealed, which have guiding significance for the study of the adsorption of different gases by anthracite. Moreover, the rationality of the model was proven from the perspective of the internal pore structure of the coal body. In addition, during the gas adsorption experiment on the coal conducted using the volumetric method, to obtain the pore volume inside the coal sample, the He gas was usually used for the testing. Since the kinetic radius of the He gas is 0.031 nm, which is less than those of CO_2_, CH_4_, and N_2_, the pore volume that the He gas can detect and enter is greater than the pore volumes that CO_2_, CH_4_, and N_2_ can detect and enter. This caused the amount of gas adsorption obtained in the experimental results to be lower than the true level. However, it can be calculated using the pore structure of the macromolecular model of coal. For example, the volume of smokeless micropores measured using the He gas in this study was 4487.71 Å^3^/g, and the volume of micropores detected using CH_4_ was 1867.55 Å^3^/g, with a difference of 2620.16 Å^3^/g. Therefore, this method can be used to assist the experiment and make up for the deficiency of the experiment, which can be used to improve the accuracy of the evaluation of coalbed methane reserves.

5. Please add error bars to all relevant figures to indicate data variability and improve result interpretation.

Response: Thank you to the reviewers for asking questions about the error bars in the graph. I agree with your views and have made many attempts. However, because most of the curves in the graph are dense, the increase of error bars leads to the overlap of the curves. Because the fitting curves in this paper are all above 0.99, the fitting effect is good, and the data error beyond 0.99 has little effect on the content of the analysis in this paper. Therefore, I suggest retaining the analysis method of the original graph, hoping to get your consent. I agree with the method of using error bars to process data, and I will use this method to process data in subsequent studies.

6. The manuscript focuses on anthracite, but the results would be more impactful if they were compared with data from other types of coal, such as bituminous or lignite. A brief comparative discussion would help place the findings in a broader context and highlight the specific advantages or disadvantages of using anthracite in adsorption processes.

Response: Thanks to the research methods proposed by the reviewers, I think it is very meaningful to analyze coal samples of different coal ranks. However, although anthracite is used as the research object in this paper, the focus is on its columnar structure, which can be maintained. Similar to the properties of coal in the ground, it is more accurate than the analysis of pulverized coal. The advanced point of the coal rock adsorption test platform under the multi-physical field conditions used in this paper is that the coal with columnar structure can be used, and the coal seam structure can be simulated by applying axial pressure and confining pressure. However, the method has commonality, and different coal types can be studied by the method provided in this paper, and relevant conclusions can be obtained. Therefore, I will take the adsorption research of various coal rank coals proposed by the reviewer as the focus of future research. At the same time, I also increase the prospect of this aspect in the conclusion part.

Conclusion:
1. The conclusion effectively summarizes the key findings but could be expanded to include a discussion on the study's limitations and potential areas for future research. For example, how might the adsorption characteristics differ with other coal types or under different environmental conditions?
2. The conclusion should also address the practical implications of the study. How can these findings inform the design of industrial processes for methane recovery or CO₂ sequestration?
3. The final paragraph could benefit from a brief mention of the broader significance of the study in the context of energy and environmental sustainability.

Response: Thank the reviewers for the guidance of the conclusion part of the manuscript. Through your guidance, I realized the shortcomings of the writing techniques in the conclusion part of the manuscript. Through your guidance, the conclusion part can more clearly reflect the research value of this paper, and the research conclusion has been sublimated. In the conclusion part, I increase the research significance and prospect of this paper. As shown below :

The methodology employed in this study can be applied to the investigation of the adsorption characteristics of other coal types, thereby offering a novel perspective on the adsorption characteristics of coal in diverse environmental contexts. The findings offer a molecular-level technical foundation for the design of industrial processes for methane recovery and CO₂ storage. It provides a theoretical foundation for the sustainable development of energy and the environment.
